# A Study on Graph Optimization Method for GNSS/IMU Integrated Navigation System Based on Virtual Constraints

**DOI:** 10.3390/s24134419

**Published:** 2024-07-08

**Authors:** Haiyang Qiu, Yun Zhao, Hui Wang, Lei Wang

**Affiliations:** 1 School of Naval Architecture and Ocean Engineering, Guangzhou Maritime University, Guangzhou 510725, China; heu_wanghui@126.com; 2 School of Automation, Jiangsu University of Science and Technology, Zhenjiang 212003, China; 211210301125@stu.just.edu.cn; 3 State Key Laboratory of Information Engineering in Surveying, Mapping and Remote Sensing, Wuhan University, Wuhan 430072, China; lei.wang@whu.edu.cn

**Keywords:** graph optimization, GNSS/IMU integrated navigation, Kalman filter, SLAM

## Abstract

In GNSS/IMU integrated navigation systems, factors like satellite occlusion and non-line-of-sight can degrade satellite positioning accuracy, thereby impacting overall navigation system results. To tackle this challenge and leverage historical pseudorange information effectively, this paper proposes a graph optimization-based GNSS/IMU model with virtual constraints. These virtual constraints in the graph model are derived from the satellite’s position from the previous time step, the rate of change of pseudoranges, and ephemeris data. This virtual constraint serves as an alternative solution for individual satellites in cases of signal anomalies, thereby ensuring the integrity and continuity of the graph optimization model. Additionally, this paper conducts an analysis of the graph optimization model based on these virtual constraints, comparing it with traditional graph models of GNSS/IMU and SLAM. The marginalization of the graph model involving virtual constraints is analyzed next. The experiment was conducted on a set of real-world data, and the results of the proposed method were compared with tightly coupled Kalman filtering and the original graph optimization method. In instantaneous performance testing, the method maintains an RMSE error within 5% compared with real pseudorange measurement, while in a continuous performance testing scenario with no available GNSS signal, the method shows approximately a 30% improvement in horizontal RMSE accuracy over the traditional graph optimization method during a 10-second period. This demonstrates the method’s potential for practical applications.

## 1. Introduction

The Global Navigation Satellite System (GNSS) plays a crucial role in technologies like unmanned driving and Simultaneous Localization and Mapping (SLAM) [1]. GNSS, which includes systems like GPS, GLONASS, Galileo, and BeiDou, provides accurate global positioning but is susceptible to signal blockages and multipath effects, particularly in urban environments. Conversely, Inertial Measurement Unit (IMU), composed of accelerometers and gyroscopes, offers high-frequency motion sensing without relying on external signals but suffers from drift over time due to sensor inaccuracies. The synergistic combination of GNSS and IMU leverages the long-term accuracy and global coverage of GNSS with the high update rate and short-term precision of IMU. This integration can be achieved through various methods, including loose coupling, where GNSS and IMU data are combined at the position level; tight coupling, which integrates data at the measurement level for enhanced accuracy during partial GNSS outages; and ultra-tight coupling, which integrates IMU data directly into GNSS signal tracking loops for maximum robustness.

GNSS/IMU integration is widely applied in autonomous vehicles, UAVs, and mobile mapping to enhance navigation stability and precision. Despite these advances, challenges such as mitigating GNSS vulnerabilities to jamming and addressing the cumulative errors in IMUs remain areas of active research and development. For GNSS receivers, despite typically receiving signals from more than four satellites in most scenarios, urban environments pose challenges due to obstructions or non-line-of-sight factors, even resulting in positioning errors of tens of meters or positioning failure [2]. Additionally, conventional outlier detection may impair satellite availability and raise the risk of an insufficient number of satellites.

In previous data processing approaches, Kalman filtering (KF) stands as a cornerstone in state estimation both in the GNSS positioning or GNSS/IMU integration system. KF is a recursive algorithm that excels in real-time applications due to its computational efficiency. It operates by predicting the system’s state and updating this prediction with new measurements, assuming Gaussian noise and linear or mildly nonlinear dynamics. In a straightforward GNSS model, its state prediction equation relies on recursive assumptions from the previous epoch. For instance, in surveying applications, it assumes constancy in the positions of measurement points, while for mobile carriers, it commonly adopts either a constant velocity model or dead reckoning assumptions [3]. Filtering incorporates historical prior information encapsulated within the previous epoch’s state, which is subsequently propagated to the current epoch through the models’ state transition equation. By merging this prior knowledge with present measurement information, an optimal estimation outcome is achieved [4,5]. However, due to uncertainties in practical applications, recent data processing methods have been leaning towards optimization theory, especially graph optimization (GO) theory.

Filtering theory faces the challenge of poorer estimation performance when incorporating interfered measurement data [6]. For example, in the current period, some measurement information may be corrupted by interference. Since the filtering process only considers the previous estimated value and the current measurement, the estimate for the current time will show significant bias [7]. The biased results caused by interference are then propagated as predictions for future processes. Even if subsequent measurements are not affected by interference, these biased results require many iterations to be corrected.

**Figure 1:**
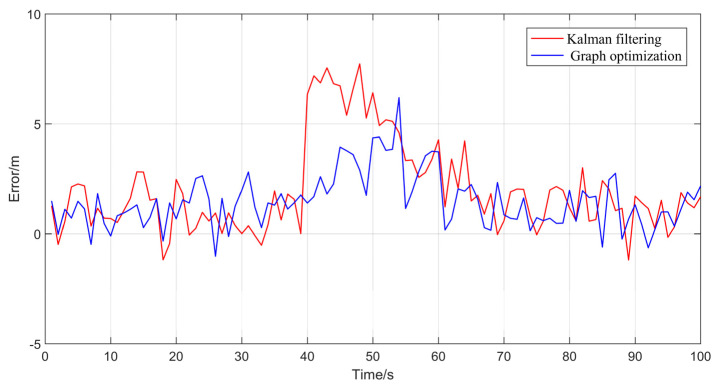
Advantages of graph optimization for handling outliers.

GO theory is a promising direction for resolving these issues. It represents the state estimation problem as a graph. In the graph, variables are nodes, measurements are edges, also referred to as constraints on the nodes, and the structure of the graph indicates their relationships. GO performs batch optimization over these graphs, considering all available data to produce a more globally consistent solution. This approach is particularly advantageous for handling a broader range of measurements, leading to more accurate and reliable state estimates. Unlike the KF, which estimates only the current state, GO can optimize the entire trajectory, making it highly effective for applications requiring high precision and robustness, although at the cost of extra computation. This concept is illustrated in Figure 1, where in the graph optimization models, an interfered measurement is just one edge among many, and this impact is distributed among all the nodes, so its effect on the single interfered node is weaker than in KF. Additionally, the number of nodes involved in the estimation is determined by a sliding window, such as sequences from 1 to 10, 2 to 11, and so on. When the affected nodes move out of the sliding window over time, they no longer impact subsequent estimates.

In recent years, there have been many cases of applying GO technology to the GNSS positioning and navigation field. A multi-sensor fusion graph optimization model based on GNSS, INS, and LiDAR has been proposed and compared with Google’s Cartographer [8]. Results from land vehicle tests demonstrate significant enhancements over traditional GNSS/INS methods. Additionally, the graph optimization model has been consistently compared with the Kalman filter method [9], highlighting the robust estimation through multiple iterations and better exploration of time correlation between measurements and states. In the real-time kinematic (RTK) field, the superiority of the graph optimization method has also been demonstrated [10]. By effectively utilizing pseudorange, carrier-phase, and Doppler measurements, this method improves positioning accuracy compared to filtering-based estimators. In the realm of real-time data processing and 3D mapping, the graph optimization method has been proven to significantly reduce errors. Evaluations in Hong Kong urban areas demonstrate substantial error reduction using this approach [11]. To address the degradation of GNSS signals caused by the urban canyon effect, the use of factor graphs and robust optimization techniques for GNSS data processing is investigated [12]. Additionally, the open-source library GTSAM is utilized to accelerate the solution process, significantly enhancing the practical applicability of the graph optimization method. Additionally, the factor graph optimization method has demonstrated high reliability in addressing outlier issues caused by multipath effects and NLOS scenarios [13,14]. In real-time vehicle positioning and mapping scenarios [15], the graph optimization method has achieved promising results by integrating information from different sensors.

The degradation of pseudorange measurement quality or the inability to measure pseudorange due to signal loss typically occurs in urban canyon environments. Currently, it is impossible to provide an accurate statistical probability for this situation because it depends on the vehicle’s path, the surrounding building environment, and road conditions. However, in urban navigation scenarios, the probability of GNSS signal degradation or loss caused by tunnels or high-rise building obstructions can reach 30% to 40%. Considering the continuity of satellite reception data across epochs, is it possible to leverage information from previous historical epochs to enhance positioning accuracy? In scenarios where real measured pseudorange information is unavailable at the current epoch, can a virtual constraint be established from the historical ephemeris data to maintain the integrity of the graph optimization model?

Based on a review of pioneering work, an initial summary can be derived: no matter whether in the GO model of GNSS/IMU, where nodes are constrained by IMU [16], or a single satellite positioning model, where nodes are constrained by constant velocity [17], the pseudorange measurements both act as edges on single epoch nodes. By adjusting the receiver’s positions along the trajectory, it is possible to minimize the overall pseudorange measurement errors, given that satellite positions remain fixed. This raises an important question: Can a satellite’s position at a specific moment be utilized by multiple pose points, thereby enabling these pose points to reuse the satellite position information? Motivated by this inquiry, this paper investigates a graph optimization method that constructs constraints using virtual pseudoranges. These virtual pseudoranges act as virtual constraints within GO, enhancing the system’s ability to accurately estimate nodes’ positions by reusing satellite information across different pose points. The contributions are primarily in three areas:

1. A method to establish virtual pseudoranges from previous satellite positions is proposed, and a corresponding GO model is developed when these virtual constraints are injected.
2. A comparative analysis between the proposed model and GNSS/IMU and SLAM models is conducted. Additionally, the differences in the marginalization processes among these models are explored.
3. Real-world GNSS/IMU data were used to conduct performance tests in both instantaneous and continuous scenarios. The pseudoranges were artificially replaced with virtual constraints created using the proposed method. These results were subsequently compared against the original GO results to demonstrate the feasibility and effectiveness of the method.

## 2. Methodology

### 2.1. Virtual Pseudorange Establishment

In a GNSS/IMU GP model, each satellite-to-receiver pseudorange measurement can be considered a constraint. Typically, positioning requires measuring the pseudorange information from at least four satellites to solve for the four variables: the three-dimensional position and the receiver clock bias. When the number of satellites is inadequate, the satellite constraints for each epoch are insufficient. In such scenarios, it is common to replace an unknown variable with a constant value, usually the clock bias. Under such operations, the remaining pseudoranges will also be affected by this fixed clock bias. Additionally, if the number of satellites is fewer than three, this method will fail.

**Figure 2:**
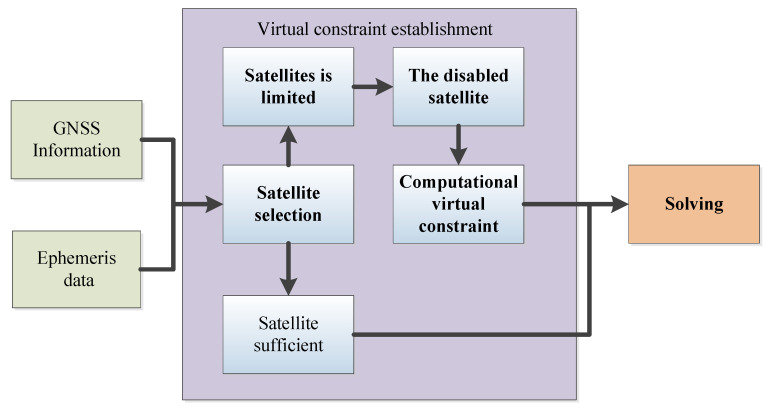
Flowchart for creating virtual constraints.

Indeed, since satellites are in motion, there wouldn’t exist two pseudorange measurements from different epochs targeting the same satellite position, resulting in two measurement constraints. Considering two consecutive epochs with a time interval of Δt, during the first epoch, a suite of data comprising the satellite’s position, pseudorange, and pseudorange rate is acquired. It is assumed that the satellite’s position at the second epoch can be extrapolated based on the previous satellite’s position and pseudorange rate, which is a strong assumption. This design process is illustrated in Figure 2.

Measured pseudorange can be expressed as:

(1) $$\rho_{i}=\left|p_{i}-p_{u}\right|+b+\mu_{\rho_{i}}$$

$\rho_{i}$ is pseudorange of satellite *i*, $p_{i}$ is position of satellite *i* during signal transmission, $p_{u}$ is position of the receiver during signal reception, b is clock difference of the receiver, and $\mu_{\rho_{i}}$ is cumulative errors of this satellite, which are all factors to consider.

While the pseudorange rate can be expressed as:

(2) $${\dot{\rho }}_{i}=\left(v_{i}-v_{u}\right)\frac{p_{i}-p_{u}}{\left|p_{i}-p_{u}\right|}+B+\mu_{{\dot{\rho }}_{i}}$$

$v_{i}$ and $v_{u}$ are the velocities of the i-th satellite and receiver, respectively, expressed in ECEF coordinate system. B indicates receiver clock drift. $\mu_{{\dot{\rho }}_{i}}$ is the set of all errors. For the same satellite, the subsequent virtual pseudorange approximation can be derived from the previous pseudorange at time t and its rate, as shown in Equation (3).

(3) $${\hat{\rho }}_{i}^{k+1}=\left[\left({\hat{\rho }}_{i}^{k}+{\hat{\dot{\rho }}}_{i}^{k}*\Delta t\right)+\left(P_{\mathrm{sat}}^{k+1}-P_{rec}^{k}-v\Delta t\right)\right]/2$$

The variable k represents the current time, while Δt denotes the duration required to transition from time k to time k+1, $P_{\mathrm{sat}}^{k+1}$ is the position of satellite at time k+1, $P_{car}^{k}$ is the position of receiver at time k, and v is the velocity of receiver. The virtual pseudorange is composed of a weighted average of two parts. One part is extrapolated based on the pseudorange and pseudorange rate from the previous epoch. The other part is derived from the difference between the satellite position calculated from the ephemeris and time t+Δt at epoch k+1 and the extrapolated receiver position at epoch k+1.

**Figure 3:**
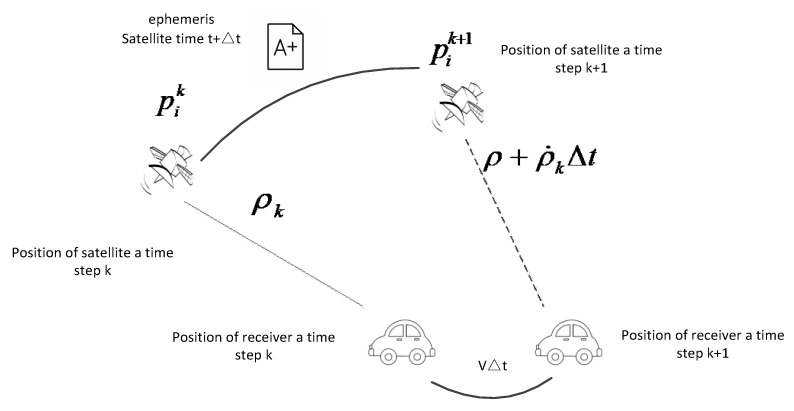
Pseudorange prediction with previous satellite position and pseudorange rate.

In high dynamic environments, such as on high-speed trains or airplanes, the pseudorange variation rate can reach several hundred (usually smaller than 500) meters per second. The pseudorange and pseudorange rate changes of a GPS receiver become more drastic and unstable. Specifically, during high-speed movement, the receiver experiences greater Doppler shifts and signal attenuation, leading to increased inaccuracies in pseudorange measurements. Therefore, continuing to use the pseudorange rate from the previous epoch in the subsequent epoch is a weak assumption. In medium to low dynamic environments, such as moving vehicles, the pseudorange variation rate is at most tens of meters (generally less than 50 m). The changes in pseudorange are relatively smooth, and significant jumps in pseudorange due to large changes in motion state typically do not occur. Therefore, it is reasonable to infer the current pseudorange based on the pseudorange and carrier’s motion state from the previous moment, as shown in Figure 3.

### 2.2. Graph Model Construction with Virtual Constraints

**Figure 4:**
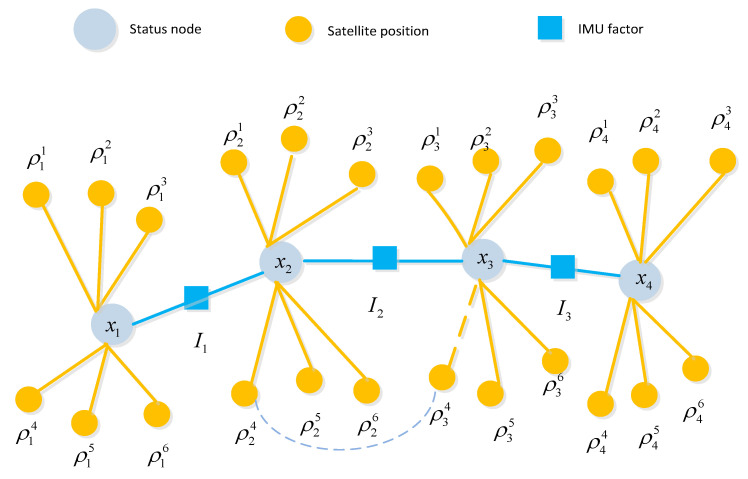
Virtual constraints-based GP model diagram for GNSS/IMU.

A complete GNSS/IMU graph optimization model consists of IMU factors, pseudorange factors, and the state (position) of the carrier, which is depicted in Figure 4. Gray nodes represent each epoch, also known as status nodes. Yellow nodes represent satellite positions, and yellow lines represent each pseudorange; solid lines represent real measured pseudoranges, while dashed lines represent constructed virtual pseudorange. The satellite position inferred from the previous moment is connected by a blue dashed line, such as $\rho_{3}^{4}$ is inferred based on $\rho_{2}^{4}$. The IMU factors accomplish motion constraint measurements between two adjacent nodes of the carrier, while the pseudorange factors provide absolute measurement constraints on the carrier’s position at a given moment.

#### 2.2.1. IMU Factor

Without considering the Earth’s rotational angular velocity and assuming the local gravitational field is constant. The angular velocity and acceleration measurement model of the IMU is as follows:

(4) $$\begin{array}{l} {\tilde{\omega }}_{b}(t)=\omega_{g}(t)+b_{g}(t)+\eta_{g}\left(t\right)\\ a_{b}(t)=\hat{R_{n}^{b}}\left(a_{n}-g_{n}\right)+b_{a}(t)+\eta_{a}\left(t\right) \end{array}$$

where ${\tilde{\omega }}_{b}\left(t\right)$ and $\omega_{g}\left(t\right)$ are measured values and real values in the carrier coordinate system, respectively, $\eta_{g}\left(t\right)$ represents the gyroscope noise, and $b_{g}\left(t\right)$ represents the zero bias of the gyroscope; $a_{b}\left(t\right)$ represents the acceleration in the carrier coordinate system, and $\hat{R_{n}^{b}}$ is the state transition matrix. $a_{n}$ represents the true value of acceleration; $g_{n}$ is the gravity vector; $b_{a}\left(t\right)$ represents the zero bias of acceleration; and $\eta_{a}\left(t\right)$ represents the noise of acceleration. The differential equation of IMU kinematics can be obtained as follows:

(5) $$\hat{{\dot{R}}_{b}^{n}}=\hat{R_{b}^{n}}\times \left(\omega_{b}\times \right)\;\;{\dot{v}}_{n}=a_{n}\;{\dot{p}}_{n}=v_{n}$$

Since the derivative of the rotation matrix is represented in exponential form, its discrete form is derived from Euler integral:

(6) $$\begin{array}{l} \hat{R_{n}^{n}}(t+\Delta t)=\hat{R_{n}^{t}}\left(t\right)\times exp\left(\omega_{h}\left(t\right)\times \Delta t\right)\\ v_{n}(t+\Delta t)=v_{n}(t)+a_{n}\left(t\right)\times \Delta t\\ p_{n}(t+\Delta t)=p_{n}(t)+v_{n}\left(t\right)\times \Delta t+\frac{1}{2}a_{n}\left(t\right)\times \Delta t^{2} \end{array}$$

where exp represents exponential mapping. By substituting Equations (4)–(6), we obtain:

(7) $$\begin{array}{l} \hat{R_{b}^{n}}(t+\Delta t)=\hat{R_{b}^{n}}\left(t\right)\times exp\left(\left({\tilde{\omega }}_{b}\left(t\right)-b_{g}\left(t\right)-\eta_{gd}\left(t\right)\right)\times \Delta t\right)\\ v_{n}(t+\Delta t)=v_{n}(t)+\hat{R_{s}^{n}}\left(t\right)\left({\tilde{a}}_{b}\left(t\right)-b_{a}\left(t\right)-\eta_{ai}\left(t\right)\right)\times \Delta t+g\times \Delta t\\ p_{n}(t+\Delta t)=p_{n}(t)+v_{n}\left(t\right)\times \Delta t+\frac{1}{2}g\times \Delta t^{2}+\frac{1}{2}\hat{R_{b}^{n}}\left(t\right)\left({\tilde{a}}_{b}\left(t\right)-b_{a}\left(t\right)-\eta_{gd}\left(t\right)\right)\times \Delta t^{2} \end{array}$$

where $\eta_{gd}$ and $\eta_{ai}$ represent discrete forms of noise. The discrete IMU measurement model from $t_{i}$ to $t_{j}$ is as follows:

(8) $$\begin{array}{l} \hat{R_{j}}=\hat{R_{i}}\cdot \overset{j-1}{\underset{k=i}{\prod }}\exp\left(\left({\tilde{\omega }}_{k}-b_{k}^{g}-\eta_{k}^{ai}\right)\cdot \Delta t\right)\\ v_{j}=v_{i}+g\cdot \Delta t_{ij}+\overset{j-1}{\underset{k=i}{\sum }}\hat{R_{k}}\cdot \left({\tilde{a}}_{k}-b_{k}^{a}-\eta_{k}^{ad}\right)\cdot \Delta t\\ p_{j}=p_{i}+\overset{j-1}{\underset{k=-1}{\sum }}[v_{k}\cdot \Delta t+\frac{1}{2}\hat{R_{k}}\cdot \left({\bar{a}}_{k}-b_{k}^{a}-\eta_{k}^{ad}\right)\cdot \Delta t^{2}] \end{array}$$

where Δt indicates the interval between two consecutive IMU data moments:

(9) $$\Delta t_{ij}=\overset{j-1}{\underset{i=j}{\sum }}\Delta t=(j-i)\Delta t$$

The state at time j can be deduced from i through a mapping function:

(10) $${\hat{x}}_{j}=h\left(x_{i},c_{i},z_{i}^{IMU}\right)$$

where $x_{i}$ and $z_{i}^{IMU}$ represent the state value and measurement value at the moment i; $c_{i}$ represents the deviation of the inertial device; h() represents a simplified mapping function of Equation (8). Then, the error function of IMU can be expressed as the following equation, and $\Sigma_{k-1}^{IMU}$ is the IMU covariance matrix:

(11) $$∥e_{k}^{IMU}{∥}_{\Sigma_{k-1}^{IMU}}^{2}=∥x_{k}-h\left(x_{k-1},c_{k-1},z_{k-1}^{IMU}\right){∥}_{\Sigma_{k-1}^{IMU}}^{2}$$

This is the pre-integration factor of the IMU, which represents the spatial position constraints between two adjacent states. It includes the angular deviations between the two states, which ultimately manifest as spatial displacement between them. This spatial displacement is indirectly related to the pseudorange measurements.

#### 2.2.2. GNSS Pseudorange Factor

The GNSS receiver receives signals from multiple satellites at a given epoch; $p_{k,r}^{e}\left(x_{k,r}^{e},y_{k,r}^{e},z_{k,r}^{e}\right)$ is the receiver’s current position under the ECEF system, and $p_{k,n}^{e}={\left(x_{p}^{e},y_{p}^{e},z_{p}^{e}\right)}^{T}$ is the satellite’s position in the ECEF coordinate system. Then, its GNSS factor can be expressed as:

(12) $$p_{k,n}=\{p_{k,1},p_{k,2},\ldots ,p_{k,i},\ldots p_{k,n}\}$$

Then, the error equation of GNSS can be obtained from the given satellite measurement [18]:

(13) $$∥e_{k,i}^{GNSS}{∥}_{\Sigma_{k,i}^{GNSS}}^{2}=∥\rho_{p,i}-h^{GNSS,TC}\left(p_{k,i},p_{k,r}^{e},\delta_{k,r}^{\mathrm{clock}}\right){∥}_{\Sigma_{k,i}^{GNSS}}^{2}$$

In Equation (10), $\rho_{p,i}$ represents the pseudorange result calculated based on the estimated position of the carrier and the satellite’s position, while $h^{GNSS,TC}$ represents the instantaneous pseudorange information calculated using the receiver’s measurements, ephemeris data, and receiver clock bias. The virtual constraint is still established using the clock bias at the current epoch, whereas the satellite position and measurement data are extrapolated from the information of the preceding epoch.

### 2.3. Graph Model Analysis Akin to SLAM Model

In this section, the proposed graph model will be compared with traditional graph models used in SLAM. Despite certain differences, there are numerous similarities between the newly constructed graph model and those in SLAM. Graph models were first extensively researched and applied in the field of SLAM, including derivative libraries such as iSAM [19] and GTSAM [20]. Consequently, the solution and processing procedures can leverage some of the operations used in SLAM.

Firstly, in the SLAM framework, landmarks are variables subject to estimation, implying their poses are not fixed. In contrast, in typical GNSS/IMU models, satellite positions are considered fixed. However, a notable variable in the estimation is the clock bias. This can be seen as introducing uncertainty similar to the positional uncertainty of the satellite. The pseudorange is adjusted by compensating for this clock bias multiplied by the speed of light. Thus, despite satellite positions being fixed in the graph, the presence of the clock bias variable in the graph model aligns satellite positions with the role of landmarks in SLAM due to their relative influence on the carrier’s positioning being similar, i.e., the state node in the graph.

Unlike the SLAM model, where the carrier can freely adjust the distance between itself and each observed landmark—such that landmark1 can be adjusted by 0.1 m and landmark2 by 0.2 m—in the GNSS/IMU graph model, the clock bias affects the distance to each satellite equally at a given moment. Thus, when the value of the clock bias is estimated, its conversion to distance (clock bias multiplied by the speed of light) will be equivalently applied for each pseudorange at one time epoch.

In the SLAM graph, a landmark can be observed by multiple nodes, i.e., observed multiple times. However, in the GNSS/IMU model, the satellite position data observed at one moment is not utilized by another moment; hence, there is no co-observation relationship. The model proposed in this paper differs from the traditional GNSS/IMU graph optimization model. In the traditional model, the pose of the carrier at any given moment and its associated satellites are independent, meaning they are only associated with that specific moment and not with satellites from other moments. However, in the model proposed in this paper, virtual satellite positions are derived from previous moments. Therefore, when constructing such virtual constraints, there is a mapping association between one satellite (with the same PRN) at different times. Consequently, there exists a factor between these two satellite nodes, as illustrated in Figure 4, with a dash line connection. Once the virtual constraint between current and previous satellite poses is built, there will be a correlation in the information matrix, which quantifies the uncertainties and relationships between variables (nodes) and measurements (edges). Unlike the SLAM model, where the landmarks are independent, the part corresponding to landmarks in the matrix maintains a sparse diagonal characteristic.

**Figure 5:**
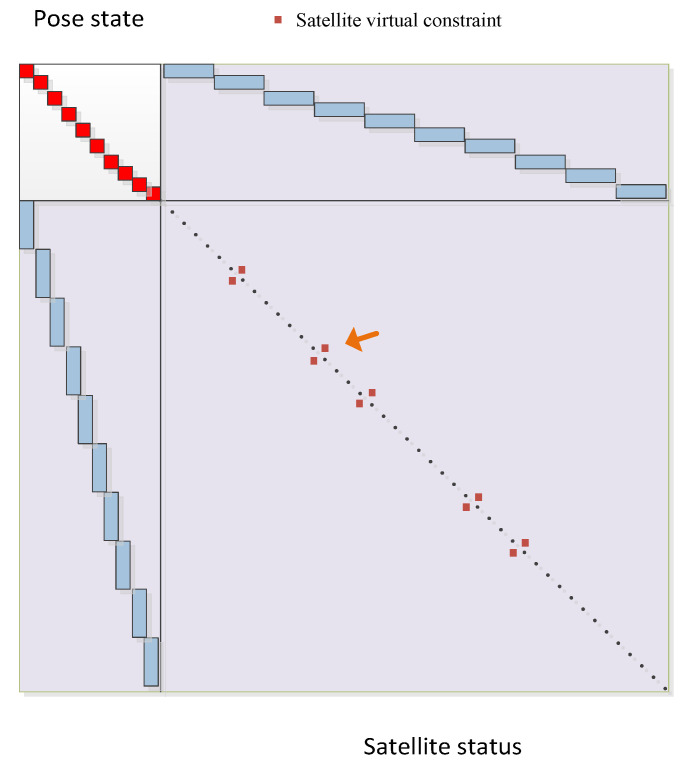
Information matrix of graph model with virtual constraints.

Analogous to the information matrix constructed by the landmark in SLAM, in the information matrix of the virtual constraint graph model, each satellite’s positions at different times with different PRNs are treated as independent node variables. These variables are uniformly placed on the right side of the information matrix. Consequently, associations between the same satellite at different times are established. However, it makes the matrix no longer purely diagonal, which is in the SLAM model, as shown in Figure 5. The red square box in the upper left corner represents the pose state of the carrier at each moment. The black dots indicate the positions of all the satellites involved in the optimization. The blue rectangular box indicates the associated satellites required for positioning at a specific moment. The orange arrows indicate that if virtual constraints are used, the information matrix is no longer a diagonal matrix.

### 2.4. Marginalization

**Figure 6:**
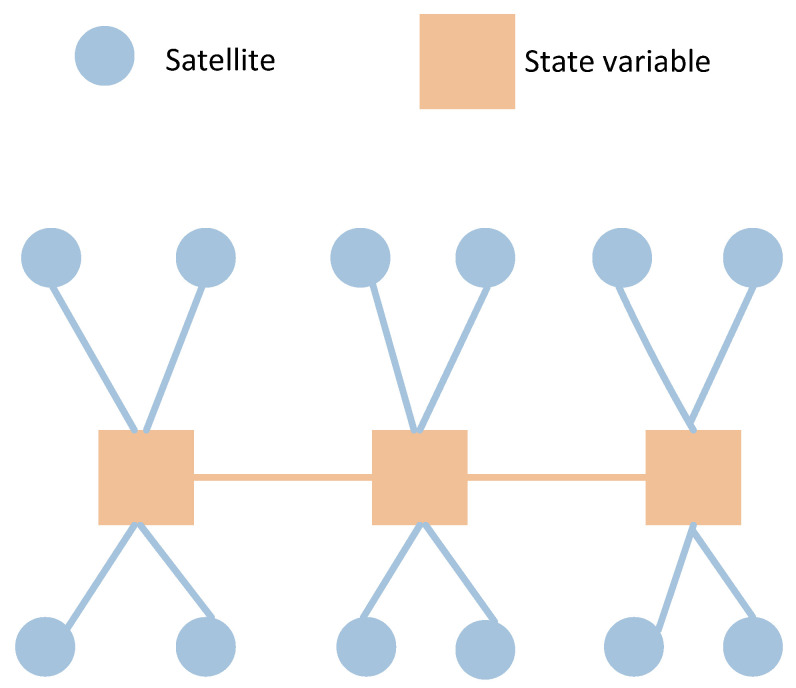
Traditional GNSS graph model.

The marginalization process in traditional GNSS graph models is quite straightforward. The satellite is isolated because the pseudorange from satellites is observed only at one state and cannot be observed in other states, as shown in Figure 6. So, if a state is prepared to be removed from the sliding window filter (SWF), it can be discarded directly without affecting the other states in the window, as this state is isolated.

**Figure 7:**
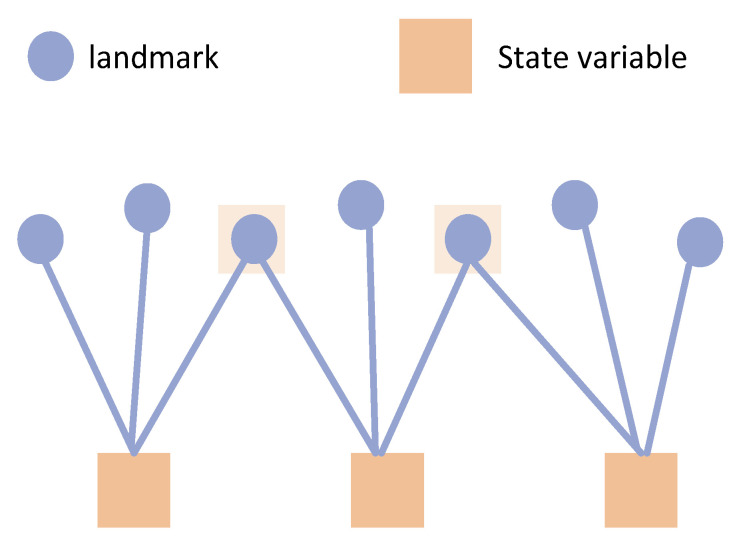
SLAM graph model.

However, in a SLAM graph model, when the first state is to be removed, it is no longer isolated due to the fact that a landmark can be observed by multiple states, as shown in Figure 7. Due to the fact that the marginalized state observed some landmarks, these landmarks gain prior information indicating where they should be given the current state of $x_{1}$.

**Figure 8:**
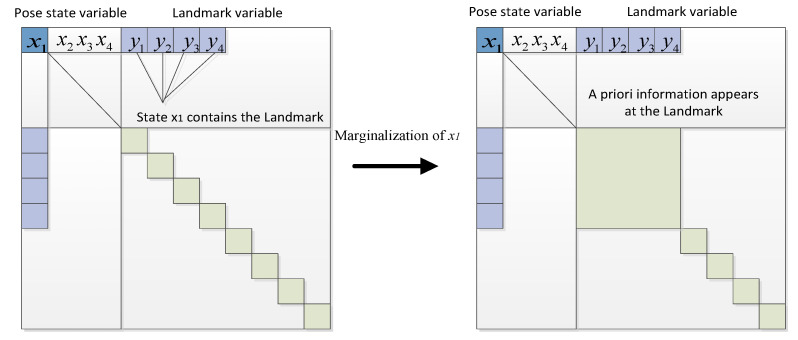
Marginalization for graph-based optimization.

Typically, this prior information is transferred to the retained states through the operation of a Schur complement. This process involves multiplying the first row of the information matrix by a coefficient and adding it to the rows below the partition line to eliminate the non-zero block in the first column. Subsequently, the first column is used to eliminate the non-zero block in the first row. However, this operation results in the Landmark–Landmark portion of the information matrix being filled with additional information, causing it to no longer remain a diagonal block, as shown in Figure 8.

**Figure 9:**
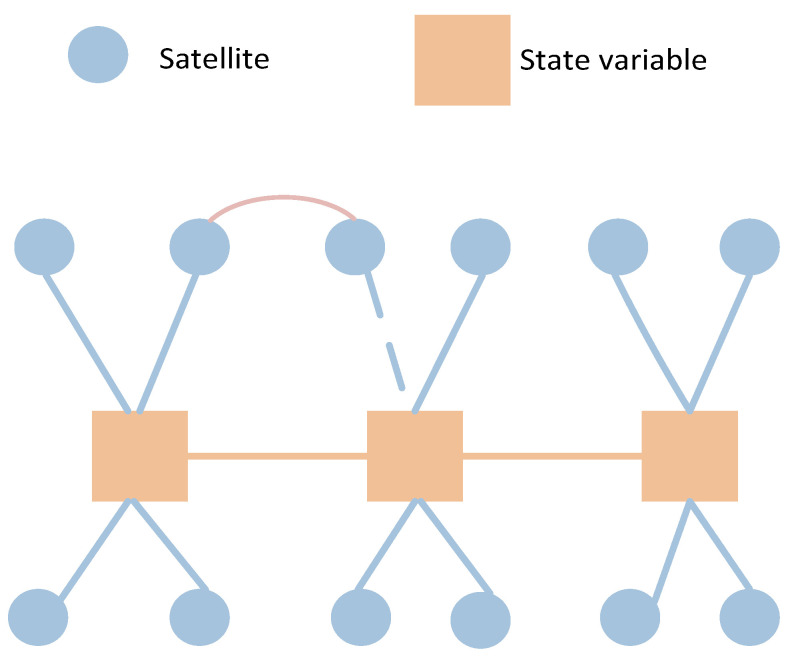
GNSS graph model with virtual constraint.

In the virtual constraint model proposed in this paper, the virtual satellite positions are constrained with associations from previous time steps. Therefore, during the marginalization process, it is necessary to simultaneously transform the priors of both the state-state and Satellite–Satellite sections, as shown in Figure 9. The positions of the satellites are determined through ephemeris and satellite time. In fact, if accurate time information is available, the satellite positions can be considered precise. Thus, the mapping error from one time step to another essentially represents the temporal error between the two epochs. However, satellite motion is nonlinear, so it is difficult to represent the mapping with an analytical function that uses time as a variable.

Therefore, in the marginalization process of this model, the state and corresponding observed satellite position information from the previous epoch will be removed. The prior for the satellite positions that need to establish virtual constraints is treated as an uncertainty, which is separated from the overall error minimization model, as shown in Equation (14). The covariance for this part is set based on the accuracy of the receiver’s oscillator.

(14) $$E_{k}=∥e_{k,i}^{GNSS}{∥}_{\Sigma_{k,i}^{GNSS}}^{2}+∥e_{k}^{IMU}{∥}_{\Sigma_{k}^{IMU}}^{2}+∥e_{k,i}^{VC}{∥}_{\Sigma_{k,i}^{GNSS}}^{2}$$

## 3. Experiments and Results

**Figure 10:**
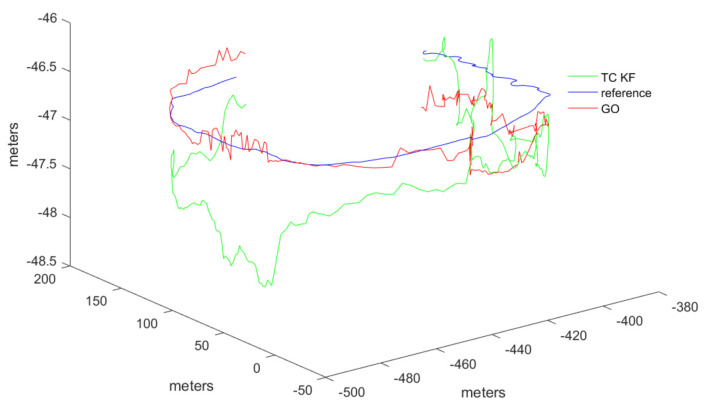
Trajectories of tightly coupled Kalman filter, GO, and reference ground truth.

To better validate the algorithm’s effectiveness, MATLAB 2019 was utilized for evaluation. As computational efficiency issues have not yet been considered, classic computational libraries like g2o and GTSAM were bypassed. To validate the proposed method’s effectiveness, the paper employs a tightly coupled open-source framework [21], tested using a set of handcart experiment data collected in Xuzhou City, China. The dataset includes reference solutions outputted by the Inertial Explorer software 9.0 in IMU/RTK mode, preprocessed GNSS observations, preprocessed IMU observations, and dual GNSS antenna orientation data. The hardware parameters are configured in Table 1 as follows:

The output results from the commercial software Inertial Explorer are used as the reference trajectory. Initially, the trajectory calculation results of the GO method and the KF method from the open-source framework were tested. The results are shown in Figure 10.

**Figure 11:**
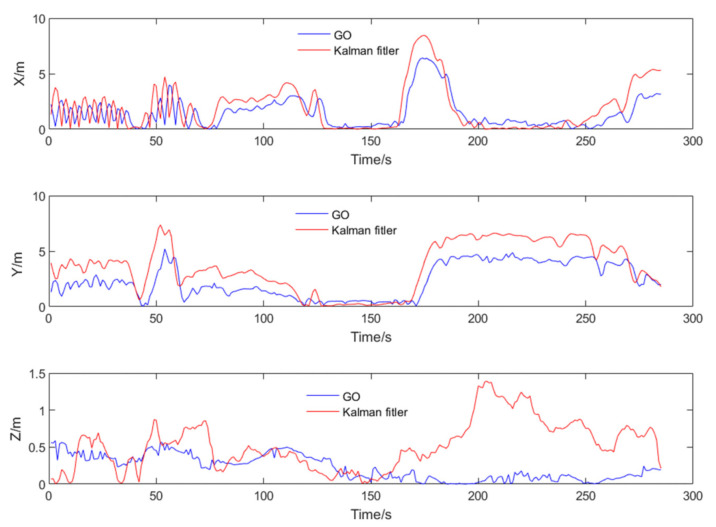
The absolute errors for GO and Kalman filter compared with reference trajectory.

To better validate the trajectory error comparison, the adopted comparison method includes the error in each direction of the carrier coordinate system, as well as the overall trajectory’s Root Mean Square Error (RMSE). RMSE provides a more intuitive measure of the overall trajectory error. Compared to the reference trajectory, the tightly coupled Kalman filtering method exhibited greater fluctuations and was more directly influenced by measurement values. In contrast, the GO method demonstrated better stability in trajectory tracking. The error trends for the three axes are displayed in Figure 11.

It should be noted that the vertical fluctuations in the Z-axis of the trajectory shown in the figures appear large due to the different scale ratios used for display. This was done to better illustrate the comparative differences in the trajectories. However, the overall trend of the trajectory distribution remains unchanged. Additionally, to provide a more direct comparison of errors, the results of both methods were statistically compared with the reference trajectory. The error statistics, which contain the three RMSE directions, are presented in Table 2.

The numerical results indicate that compared to the KF results, GP significantly improved estimation accuracy in terms of RMSE, with error reduction in all three directions close to 30%. The main reason is that the test trajectory showed a consistent pattern, with the vehicle moving in a circular motion on a flat surface. GNSS positioning results often exhibit large jumps, and these results, when used as measurements in KF, negatively impact the final KF estimates. Since GP uses a batch estimation method, it demonstrates good stability and estimation accuracy when the motion pattern is consistent, and the state of motion does not change significantly. Additionally, the horizontal accuracy of GNSS-only positioning results is typically better than vertical accuracy. However, the results in Table 2 contradict this because they incorporate IMU information, and the vehicle’s movement in the gravitational (vertical) direction is minimal, resulting in smaller vertical errors in the statistical results.

**Figure 12:**
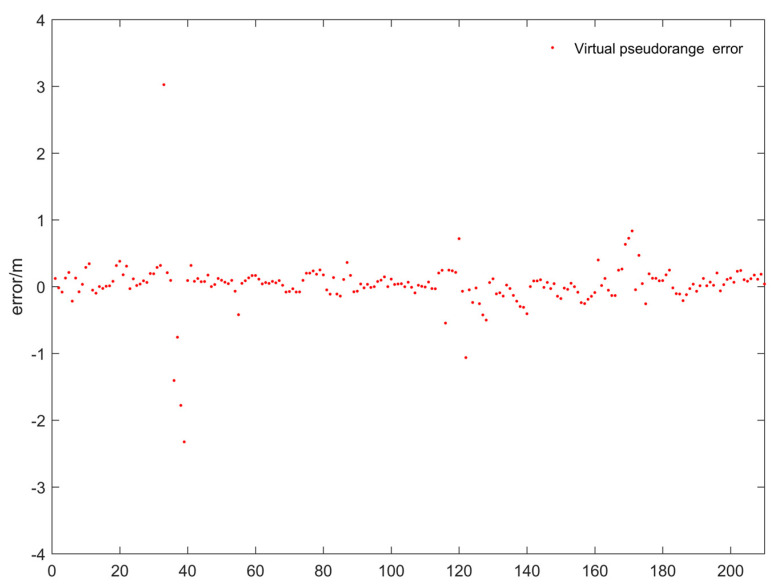
The error between virtual pseudorange and real measured pseudorange.

To verify the actual performance of the virtual constraints and determine its impact on navigation system error, positioning results with and without the virtual constraints were compared, and an error analysis was conducted. First, the difference between the virtual and real pseudoranges was validated by comparing the virtual pseudorange with the measured pseudorange. In the test data, 213 pseudorange measurements were randomly selected. The pseudorange for the next time step was constructed using the method in 2.1 and compared with the real pseudorange measured at that next time step. The error results are shown in Figure 12.

**Figure 13:**
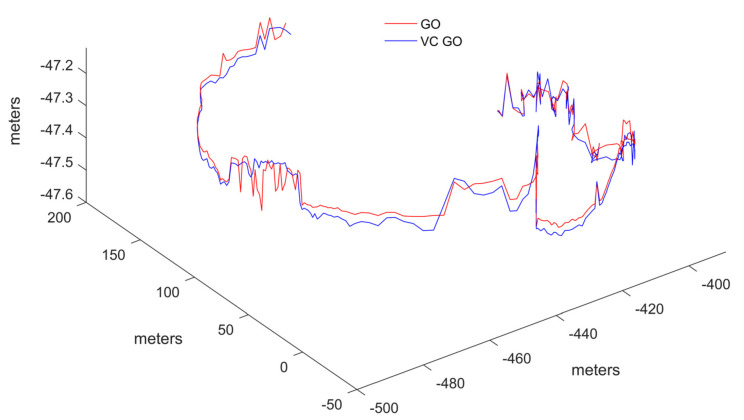
Trajectories of GO and VC GO using test data.

The error trend shown in the figure indicates that most of the errors between the virtual and real pseudoranges are within 1 m, with a maximum error of 4 m in the test data. When these virtual pseudoranges are injected into the graph model, replacing the original real pseudoranges, the impact on positioning differences is shown in Figure 13. The numerical differences in the final positioning results are significantly smaller compared to the differences between the GO results and the KF results. To highlight the comparison details, the trajectory only displays the comparison between the GO with virtual constraints (VC GO) and the original GO because including the reference trajectory would show even closer alignment due to scale. From a trend perspective, VC GO exhibits good consistency.

**Figure 14:**
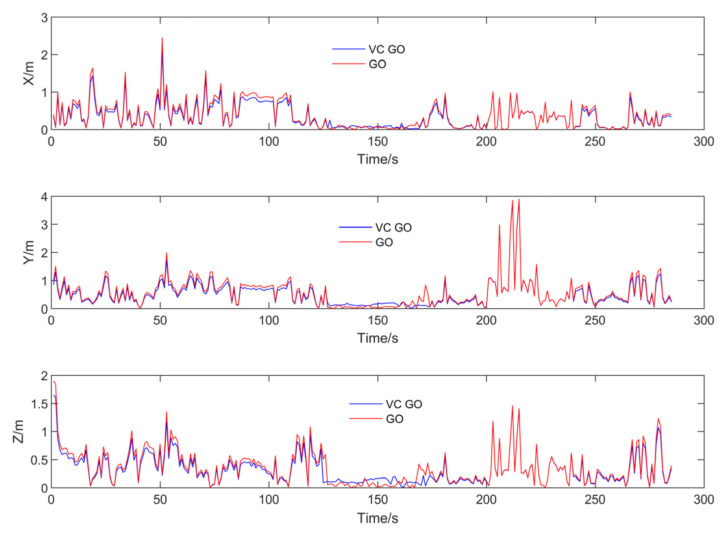
The error between GO and VC GO in three-axis directions.

Similarly, to better quantify the positioning differences between the two methods, the values in the three-axis directions were compared with the reference trajectory as shown in Figure 14.

Similarly, the RMSE errors and error variation rate for the three axes are shown in Table 3.

The error distribution trend and statistical results indicate that the positioning result differences between VC GO and GO are not significant. The RMSE error in the horizontal axes is within 2%, and in the vertical Z-axis, it is within 5%. In addition to instantaneous performance comparison tests, this paper also considers the method’s performance in continuous application tests. Considering the situation where GNSS positioning is not feasible due to insufficient satellites, GO will rely solely on IMU factor constraints. In other words, IMU results may diverge without correction. In this scenario, the paper compares the results of VC GO with GO results that do not involve GNSS positioning. Considering urban tunnel conditions where the vehicle might not receive any GNSS signals, VC GO uses four virtual pseudoranges to continue simulating positioning results. In the test data, 20 starting points were randomly selected. The graph optimization estimation results were tested at 5 s and 10 s after the starting points, and the RMSE errors of the three axes were averaged. The 10-second period allows navigation through most short tunnels and obstructing buildings in urban road conditions. Again, the RMSE errors of VC GO and GO, both compared to the reference trajectory, are shown in Table 4.

The experimental results indicate that in the absence of real GNSS measurements, both estimation methods showed degradation in accuracy. However, the results using virtual pseudorange constraints were significantly better than those using only IMU estimation, demonstrating strong error suppression capabilities. Particularly in the horizontal direction, for example, with a 10-second period, the estimation accuracy improved by approximately 30%. In the vertical direction, the improvement was less than 10%. This situation arose because the test trajectory exhibited more changes in the horizontal direction while the vertical component remained relatively consistent. Therefore, the IMU’s vertical component variation was minimal, whereas the horizontal errors diverged due to changes in heading. However, in all three axial directions, VC GP maintained better estimation accuracy.

## 4. Conclusions

The virtual pseudorange constraint graph optimization method proposed in this paper can effectively improve positioning accuracy when GNSS pseudorange measurement accuracy is degraded due to multipath or NLOS conditions or when the number of satellites is fewer than four. This method is designed based on a GNSS/IMU integrated navigation system. First, a method for calculating virtual pseudoranges based on the previous epoch was introduced. A graph optimization model was built by integrating virtual pseudoranges with the IMU factor. Then, the structure of the model and its marginalization were analyzed. Finally, using real-world data, three cases were tested: the KF solution method versus the GP method; the virtual constraint-based GP method versus the GP method; and the GP method versus the virtual constraint GP method in the GNSS positioning deny scenario. In the test data, the GP method showed higher estimation accuracy than the KF method, and the virtual constraint GP method had similar instantaneous estimation performance to the traditional GP method. This demonstrates the feasibility of VC GP in replacing individual pseudorange with significant errors due to short-term interference. In long-period experiments assuming a scenario where GNSS positioning is unavailable, meaning the traditional GP only included IMU factors, the VC GP model achieved better estimation accuracy, improving horizontal accuracy by about 30%. This proves that VC GP has strong error suppression capability when satellite signals are insufficient for positioning.

## Figures and Tables

**Table 1:** System hardware configuration settings.

Hardware Parameters	Value
GNSS Signal Frequency (Hz)	1
IMU Frequency (Hz)	125
Gyroscope Bias (rad/s)	0.0005
Accelerometer Bias (μg)	80
Carrier-to-Noise Ratio Threshold	30
Operation Time (s)	292

**Table 2:** The RMSE of three directions for Kalman filter and GO compared with reference trajectory.

Methods	X-Direction Error	Y-Direction Error	Z-Direction Error
Kalman filter	2.83 m	4.16 m	0.61 m
GO	1.96 m	2.64 m	0.45 m
Error reduction	30.1%	36.5%	25.8%

**Table 3:** The RMSE of three directions for VC GO and GO compared with reference trajectory.

Methods	X-Direction Error	Y-Direction Error	Z-Direction Error
VC GO	1.99 m	2.68 m	0.48 m
GO	1.96 m	2.64 m	0.45 m
Error variation rate	1.667%	1.623%	4.891%

**Table 4:** The RMSE in three directions for VC GO and GO compared with the reference trajectory over 5 and 10 s in a continuous performance test scenario.

Period	Methods	X-Direction Error	Y-Direction Error	Z-Direction Error
5 s	VC GO	2.45 m	2.99 m	0.53 m
	GO	3.10 m	3.85 m	0.55 m
	Error reduction	21.02%	22.32%	3.2%
10 s	VC GO	3.11 m	3.54 m	0.69 m
	GO	4.43 m	5.15 m	0.74 m
	Error reduction	29.73%	31.26%	6.92%

## Data Availability

The data used in this paper is available at the link https://github.com/benzenemo/TightlyCoupledINSGNSS (accessed on 24 May 2024).
